# Growth and Demography of the Solitary Scleractinian Coral *Leptopsammia pruvoti* along a Sea Surface Temperature Gradient in the Mediterranean Sea

**DOI:** 10.1371/journal.pone.0037848

**Published:** 2012-06-01

**Authors:** Erik Caroselli, Francesco Zaccanti, Guido Mattioli, Giuseppe Falini, Oren Levy, Zvy Dubinsky, Stefano Goffredo

**Affiliations:** 1 Marine Science Group, Department of Evolutionary and Experimental Biology, Alma Mater Studiorum - University of Bologna, Bologna, Italy, European Union; 2 Operative Unit of Radiology and Diagnostics by Images, Hospital of Porretta Terme, Local Health Enterprise of Bologna, Porretta Terme, Italy, European Union; 3 Department of Chemistry, University of Bologna, Bologna, Italy, European Union; 4 The Mina and Everard Goodman Faculty of Life Sciences, Bar-Ilan University, Ramat-Gan, Israel; Leibniz Center for Tropical Marine Ecology, Germany

## Abstract

The demographic traits of the solitary azooxanthellate scleractinian *Leptopsammia pruvoti* were determined in six populations on a sea surface temperature (SST) gradient along the western Italian coasts. This is the first investigation of the growth and demography characteristics of an azooxanthellate scleractinian along a natural SST gradient. Growth rate was homogeneous across all populations, which spanned 7 degrees of latitude. Population age structures differed between populations, but none of the considered demographic parameters correlated with SST, indicating possible effects of local environmental conditions. Compared to another Mediterranean solitary scleractinian, *Balanophyllia europaea*, zooxanthellate and whose growth, demography and calcification have been studied in the same sites, *L. pruvoti* seems more tolerant to temperature increase. The higher tolerance of *L. pruvoti*, relative to *B. europaea*, may rely on the absence of symbionts, and thus the lack of an inhibition of host physiological processes by the heat-stressed zooxanthellae. However, the comparison between the two species must be taken cautiously, due to the likely temperature differences between the two sampling depths. Increasing research effort on determining the effects of temperature on the poorly studied azooxanthellate scleractinians may shed light on the possible species assemblage shifts that are likely to occur during the current century as a consequence of global climatic change.

## Introduction

Climate change is the defining environmental, economic, and social issue of our time, and it is now certain that the rapid increase in CO_2_ concentration in the atmosphere since the 19^th^ century industrial revolution is driving significant changes in the physical and chemical environment of Earth [1]. The rate of global climatic changes is accelerating, and the average surface temperature of the Earth is likely to increase by 1.1–6.4°C by the end of the 21^st^ century [2]. Growing evidence suggests that climate change is having more substantial and rapid effects on marine communities than on terrestrial ones [3]. Synergy among increased seawater temperature, enhanced ultraviolet-B (UVB) radiation, surface ocean acidification, and human anthropogenic stress is affecting all levels of ecological hierarchies in a broad array of marine ecosystems [4]. The magnitude of temperature warming is expected to be greater in temperate areas than in tropical ones [2]. The Mediterranean basin is likely to be one of the regions most affected by the ongoing warming trend and by an increase in extreme events [5], thus representing a natural focus of interest for research. The Mediterranean is already one of the most impacted seas in the world, due to its central position as the cradle of civilization in antiquity, and as a contemporary hub of oil and commodities shipping [6]. The enhancement of interactions between climate change and many other disturbances such as eutrophication caused by fertilizer runoff and the damming of rivers [7] all increase the stresses to which Mediterranean biota are exposed.

Since the first investigations, the characteristics of scleractinian population structure and dynamics have been related to environmental conditions and their effects on these corals' symbiosis with unicellular algae [8]–[12]. It is now commonly accepted that the demographic traits of coral populations may reveal relationships between the organisms and their environment, and can be used to assess habitat stability and suitability [8], [13]–[16]. Moreover, information such as population turnover can be used to design strategies for reef restoration and bioremediation of degraded or damaged coastal areas [17]–[20].

Notwithstanding their importance, few studies have quantified life-history parameters of scleractinian corals, partly because of the processes of fragmentation, fusion and partial colony mortality, which cause corals of similar size to be of widely different ages, thus distorting the age-size relationships [12], [21]. The scarce studies on population dynamics of scleractinian corals were reviewed in the ‘70 s, describing their growth and survivorship [17]. Since then, demography has been studied for some species in the Southwestern Atlantic [22], Pacific [23], Red Sea, Caribbean, Great Barrier Reef, and the Mediterranean [16]. Replication, growth and death of the modules can be used to model the growth of modular individuals [24], and studies of modular growth have often focused on plasticity of form and the complexity of both individual colony growth and population dynamics of these organisms [12], [21], [25].

Coral age can be reliably determined in species whose individuals rarely fragment or fuse, and where partial mortality can be recognized by anomalies in the regular growth pattern [19], [21]. The growth and dynamics of modular organisms that fulfill these prerequisites can be analyzed using age-based models applied to colony morphology [8], [26], [27]. In some solitary corals, age estimates can be obtained from externally visible growth bands [19], [20]. Growth band analysis has been used to determine the age of colonial scleractinian and gorgonian corals [27]–[29], and in solitary forms [15], [16], [19], [20], [30]. Hence, for some species growth and population dynamic models based on age can be applied to describe demographic characteristics [15], [16], [19], [20], [26], [30], [31]. Recently, an age-based Beverton-Holt model provided an adaptive management approach for regulating an octocoral fishery for bioactive compounds in the Bahamas, avoiding long-term characterization of population dynamics which is rarely feasible [32].

**Figure 1:**
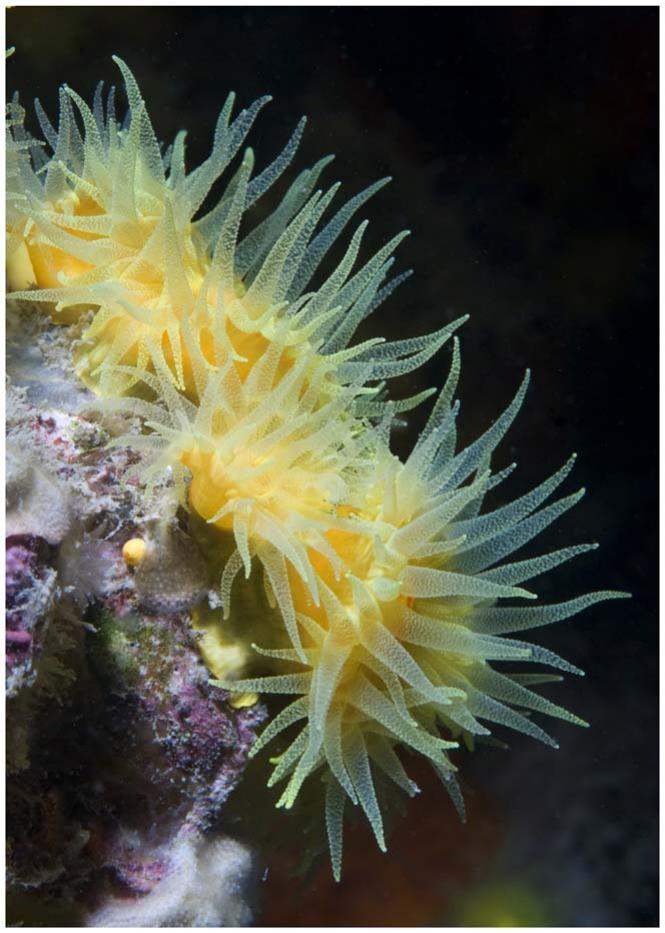
Living specimens of *Leptopsammia pruvoti* in an overhang on the Italian coasts.

*Leptopsammia pruvoti* Lacaze-Duthiers, 1897 is an ahermatypic, non-zooxanthellate, and solitary scleractinian coral, widely distributed in the Mediterranean basin and along the European Atlantic coast from Portugal through Southern England and Ireland [33]. It is one of the most common organisms in semi-enclosed rocky habitats, under overhangs, in caverns, and small crevices at 0–70 m depth [33]. Sea surface temperature (SST) and solar radiation along an 850-km latitudinal gradient in Western Italian coasts have been reported not to significantly influence neither its population abundance, nor skeletal architecture features such as corallite length, width, height [34]. However, the density of the calcium carbonate crystals of its skeleton (micro-density) [35] is positively correlated with SST [36]. It is a gonochoric internal brooder [37], with a genetic structure characterized by heterozygote deficits at all scales, from patch to populations, without correlations between genetic differentiation and geographic distance and with most genetic differentiation occurring between patches of the same study site, rather than between sites [38]. Its bright yellow colour and abundance makes this species attractive to recreational divers, who represent an important income for coastal tourist resorts in the Mediterranean [39] (Fig. 1).

The aim of this study was to determine the growth and population dynamics traits of *L. pruvoti* in sites along a latitudinal gradient spanning 850 Km along the Italian west coast and a 2°C range of average SST. Considering the detrimental population conditions reported in sites with higher SSTs for the Mediterranean solitary dendrophylliid coral *Balanophyllia europaea* studied in the same sites, during the same time interval, and using the same methods as the present study [16], [34], [36], [40], we tested the hypothesis of a negative correlation with SST for the growth and population dynamics traits of *L. pruvoti*. Ours is the first investigation of the growth and demography characteristics of an azooxanthellate scleractinian along a natural SST gradient. The counterintuitive results we obtained are considered in relation to the possible different sensitivity to global warming between corals hosting algal symbionts and those devoid of such mutualistic symbioses.

## Methods

### Sample collection

**Figure 2:**
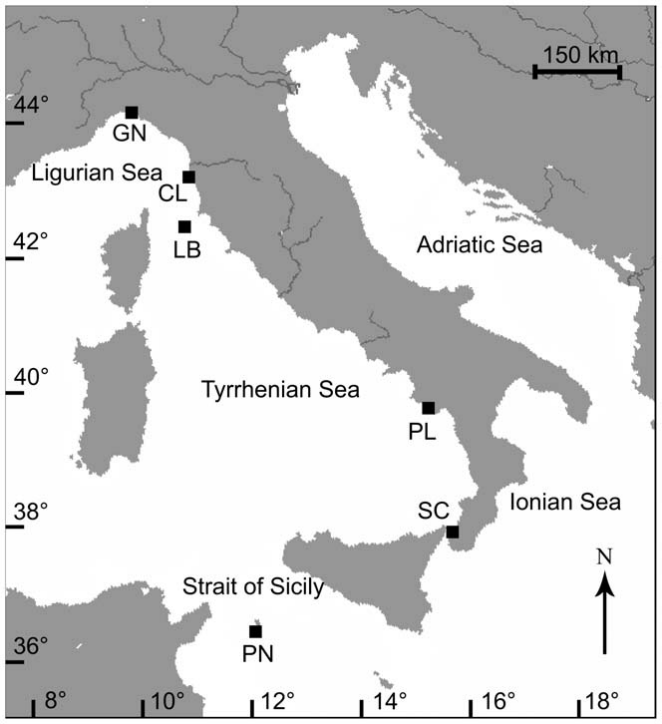
Map of the Italian coastline indicating sites where corals were collected. Abbreviations and coordinates of the sites in decreasing order of latitude: GN Genova, 44°20′N, 9°08′E; CL Calafuria, 43°27′N, 10°21′E; LB Elba Isle, 42°45′N, 10°24′E; PL Palinuro, 40°02′N, 15°16′E; SC Scilla, 38°01′N, 15°38′E; PN Pantelleria Isle, 36°45′N, 11°57′E.

From 9^th^ November 2003 to 30^th^ September 2005, specimens of *L. pruvoti* were collected from six sites along a latitudinal gradient, from 44°20′N to 36°45′N (Fig. 2). Latitude is the main factor influencing the variation in SST [41], which is the environmental parameter considered in this study and that has already shown correlations with biologic parameters of *L. pruvoti* in previous studies [34], [36]. Samples were collected for each site using transects that consisted of at least 3 triangular patches of basis*height equal to 12 cm * 7.1 cm (single patch area = 42.6 cm^2^; transect area per each site = at least 42.6*3 = at least 128 cm^2^; Table 1). A triangular shape of the patch was chosen because it was more easily placed in the narrow crevices colonized by the species, with respect to traditional square patches. Such a small patch area was chosen because of the high population density of the species (about 10000 individuals m^−1^) which makes the sampling of all individuals present in larger areas (such as 1 m^2^) unfeasible [34]. Moreover, such sampling area is considered representative of the studied site in previous studies of the biometry and growth of this species, where significant differences among sites and correlations with SST have been found [30], [34]. Patches were collected on the vault of crevices 3 m apart, at a depth of 15–17 m, during a single dive per site. Patches were located in crevices clearly separated one from each other, without a continuous presence of polyps from one patch to each other. The analysis of genetic differentiation between *L. pruvoti* populations living in different sites [38] shows that most genetic differentiation occurs between patches of the same study site, rather than between sites. Since the goal of the present manuscript was to check differences in growth and population dynamics and their relations with SST in coral populations subject to different SST regimes, to have a meaningful picture of the growth and population dynamics conditions at each site it was necessary to sample different patches at each site and treat them as replicates. Using replicates from different patches to characterize the conditions at each site, considers a wider proportion of the genotypes living in that site than by treating each patch as a separate population. Because of the random distribution pattern of the species, the problems associated with regularly spaced quadrats and transects do not apply to this study [34]. All of the polyps present in each patch were collected (See Table 1 for the number of samples collected in each site). The sampling took place at depths known to have high population densities and where the reproductive biology, biometry, population genetics and dynamics of this species had been studied previously [30], [34], [37], [38], [42].

**Table 1:** Sea surface temperature, number of patches and collected samples, *L_∞_*, *K*, *r^2^* (coefficient of determination of the semi-log regression of Eq. 2, which is an estimator of population structure stability) and demographic parameter values of each sampled populations.

Population	Genova	Calafuria	Elba	Palinuro	Scilla	Pantelleria	General
Code	GN	CL	LB	PL	SC	PN	
SST (°C), Annual Mean (SE)	19.556 (0.038)	18.023 (0.036)	18.737 (0.038)	19.138 (0.032)	19.537 (0.021)	19.875 (0.036)	
Number of patches	3	3	4	3	3	3	
Number of samples collected	123	210	76	152	115	144	
*L_∞_* (mm)	9.2	8.6	10.2	9.3	15.9	10.0	15.4
*K*	0.218	0.185	0.114	0.136	0.107	0.120	0.062
*R^2^*	0.410	0.839	0.421	0.675	0.719	0.796	-
Instantaneous rate of mortality (*Z*)	0.073	0.427	0.123	0.211	0.115	0.249	-
Observed % of immature individuals	30.9	28.6	13.2	36.8	35.7	22.9	-
Theoretical % of immature individuals	21.9	57.5	31.5	46.9	52.6	29.9	-
Observed mean age (years)	5.9	3.0	7.7	4.3	6.1	5.7	-
Theoretical mean age (years)	9.6	1.9	7.0	4.2	7.3	3.5	-
Observed age at max % biomass (years)	8	4	11	8	12	11	-
Theoretical age at max % biomass (years)	20	5	15	11	16	10	-
Observed mean age of biomass (years)	9.2	4.0	10.2	6.9	13.8	8.8	-
Theoretical mean age of biomass (years)	19.1	6.2	16.9	13.7	17.5	12.7	-

Growth data of the CL population were taken by [30]. The populations are arranged in decreasing order of latitude. SE = standard error.

### Sample analysis

All collected corals were dried at 50°C for four days and observed under a stereoscope to remove fragments of substratum and calcareous deposits produced by other organisms. Corallite length was selected as the main biometric parameter, since it is a good indicator of skeletal mass and has been used as the primary measure of size in other biometric, reproductive biology and population dynamics studies of this species and other solitary corals [15], [16], [20], [30], [34], [37], [43]–[45]. Corallite length (*L*: maximum axis of the oral disc) of all corals (see table 1 for number of corals in each site) was measured using a caliper, and corallite mass was weighed with an analytical balance [20], [44], [46].

### Growth and population demography modeling

By means of computerized tomography (CT), the number of annual growth bands was counted on about 30 skeletons randomly selected from the collected samples aimed at obtaining an objective relationship between corallite size and age. This technique is commonly applied to scleractinian corals [47], [48] and has been successfully used for *L. pruvoti* [30]. The age of each skeleton was determined from the growth-band counts, based on one high-density band in winter and a low density band in summer [30], [49].

**Figure 3:**
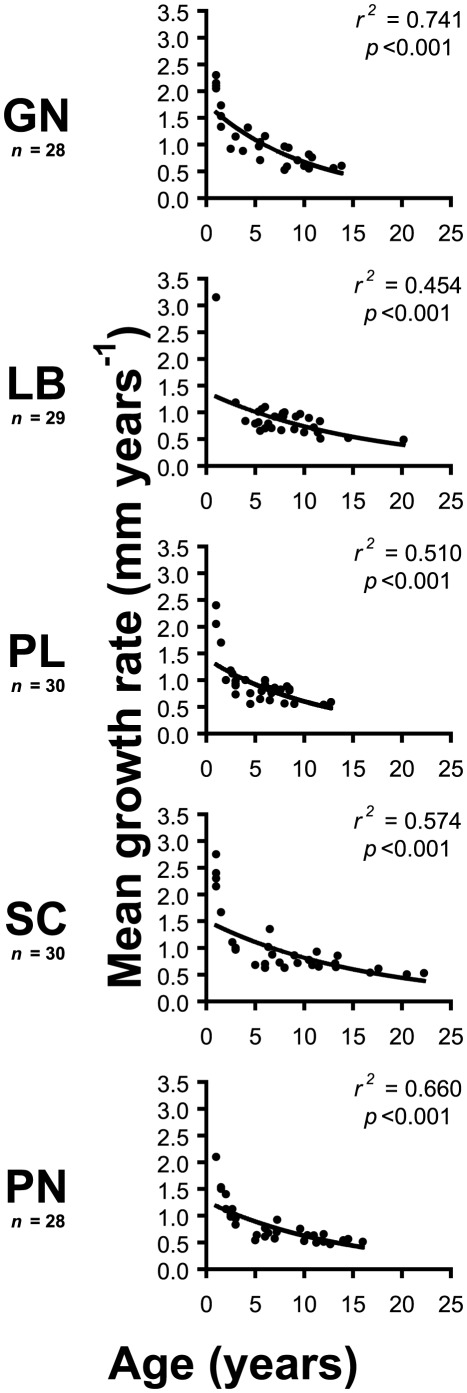
Relationships between mean growth rate and age of each population. Data were fitted with exponential curves to verify the exponential decrease of growth rate with age assumed by the von Bertalanffy growth model. *n* = number of individuals dated by computerized tomography scans (CT).

The von Bertalanffy growth model [50] predicts decreasing growth rate with age, approaching zero asymptotically, and has been validated for the Calafuria (CL) population of *L. pruvoti* using CT density bands and field measurements [30]. To test if the von Bertalanffy function could be used for all populations sampled in this study, the decreasing growth rate of *L. pruvoti* with age was checked at each location [51]. For each sample dated by CT scans, a mean growth rate was obtained by dividing length by age, and the mean growth rate was plotted against individual age (Fig. 3). All the populations showed a marked decrease of mean growth rate with age, and fitted a negative exponential curve (Fig. 3), from which growth was modeled with the von Bertalanffy function [50]:
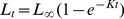
(1)where *L_t_* is individual length at age *t*, *L_∞_* is asymptotic length (maximum expected length in the population), *K* is a growth constant (higher for a fast growth up to the asymptotic length, smaller for a slow one), and *t* is the age of the individual. The parameters *L_∞_* and *K* were determined by applying the “Ford-Walford plot” method [15], [30], [52]–[55].

The population size structures were obtained from the survey transects, and age structure was determined using Equation 1. In a theoretical steady state population, 100% of the variance of the frequency of age classes is explained by age. To estimate population structure stability, the age frequency distribution was analyzed using a regression analysis of the natural logarithm of the numbers of individuals (frequency) in each age class (*N_t_*) against their corresponding age (*t*), or
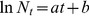
(2)The slope *a* with sign changed is usually indicated as *Z* (instantaneous rate of mortality), and can be used to estimate the theoretical numeric reduction of individuals over time, the intercept *b* is equal to the natural logarithm of the number of individuals at age zero (*N_0_*) [15], [16], [19]–[21], [30], [54], [55]. In a theoretical population in a steady state (rate of recruitment equal to rate of mortality) [26] the coefficient of determination (*r^2^*) is equal to unity [54], [56]. As natural populations deviate from the steady state, *r^2^* decreases to zero. This method for estimating population stability has previously been used for colonial and solitary corals [15], [16], [19]–[21], [26], [31], [57], including *L. pruvoti* [30].

The instantaneous rate of mortality *Z* was used to express the theoretical reduction of the corals over time (survivorship curve):
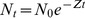
(3)
*N_t_* is the number of individuals in each age class, *N_0_* is the number of individuals at age 0, *Z* is the instantaneous rate of mortality (slope of Equation 2 with sign changed), *t* is the age.

The mean age of the individuals at each site was computed from that of samples dated with the growth curve (Equation 1). The observed percentage of individuals below sexual maturity was obtained by summing the frequencies of the age classes below sexual maturity, which is 2–3 years (3 mm length) [30], [37]. The theoretical mean age was estimated as that of the theoretical number of individuals at each site. The theoretical percentage of individuals below sexual maturity was estimated by summing the frequencies of the theoretical number of individuals of the age classes below sexual maturity at each site.

The observed biomass distribution per age class was obtained by adding the mass of each corallite in each age class. A theoretical age-mass growth curve was obtained for each site using the age-length growth curve (Equation 1) and the length-mass relationship from [34]. The theoretical biomass distribution per age class was then obtained by multiplying the theoretical number of individuals in each age class (according to the survivorship curve, Equation 3) for the expected mass at that age. The theoretical age at maximum percentage biomass was estimated as the age class representing the highest percentage biomass. The observed age at maximum percentage biomass was determined in the same way, using the observed biomass distribution. The observed mean age of biomass in the population was calculated as the sum of the products of the observed biomass in each age class multiplied by its age, then divided by the total observed biomass. This parameter estimates how old the biomass is at each site; populations with most of the biomass accumulated in younger corals will have a lower mean age of biomass than populations in which most of the biomass is represented by older individuals [16]. The theoretical mean age of biomass in each site was calculated in the same way, but using the theoretical biomass in each age class and the total theoretical biomass.

### SST data

SST data for the years 2003–2005 were obtained for each location from the National Mareographic Network of the Agency for the Protection of the Environment and Technical Services (APAT, now renamed to Superior Institute for Environmental Research and Protection, ISPRA, data available at http://www.isprambiente.gov.it). The data were from stations close to the sampling sites (<1 km) at a depth of 1 m below minimum low tide level. Mean annual SST values were computed from hourly measurements from January 2001 through January 2005 (Table 1). Three digital thermometers (i-Button, DS1921G-F5#, Maxim Integrated Products, Dallas Semiconductors) were placed close to the experimental sites of Genova, Calafuria, Scilla and Pantelleria at 16 m, to record seawater temperature every 2–3 hours during a time interval depending on the site (Figure S1). Thermometers were replaced every 3 months to avoid problems of encrustation and overgrowth by marine organisms. Thermometer data were used to check if SST data are representative of the temperature at the depth of coral sampling.

### Statistical analyses

Because of the heteroskedastic data sets, non-parametric Kruskal-Wallis was used to compare mean SST among the sites. Analysis of covariance (ANCOVA) was used to compare the slopes and intercepts of the equation of the Ford-Walford plots from which *K* and *L_∞_* values were obtained. Pearson correlation coefficients were calculated for estimating population structure stability at each site (coefficient of determination of Equation 2), and for the relationships between SST and *L_∞_*, *K*, population structure stability, observed and theoretical % of individuals below sexual maturity, observed and theoretical mean age, observed and theoretical age at maximum % biomass, observed and theoretical mean age of biomass. Because of the low *n* value (*n* = 6) and the assumptions of the Pearson method, correlation coefficients were also estimated with bootstrapping [58], with 100,000 resamples. The non-parametric Kolmogorov-Smirnov test was used to compare the age frequency distributions among sites. All analyses were computed using PASW Statistics 18.0.

## Results

The correlation between average daily SST data from data banks and average daily temperature data collected by the digital thermometers at 16 m produced *r^2^* values ranging from 0.784 to 0.935, indicating that 78–94% of the variance of seawater temperature at 16 m is explained by SST variations (Fig. S1). At Calafuria, the mean difference between SST and temperature at 16 m on an annual basis was 2.18°C (SD = 1.98°C; SE = 0.04°C). Sampling sites were characterized by significantly different mean annual SST values (Kruskal-Wallis, *p*<0.001; Table 1).

All sites were characterized by a negative exponential relationship between mean growth rate and age, with age explaining 45–74% of growth rate variance (Fig. 3). Growth rates decreased from 1–1.5 mm year^−1^ at age <5 years to 0.4–0.8 mm year^−1^ at age >10 years (Fig. 3).

**Figure 4:**
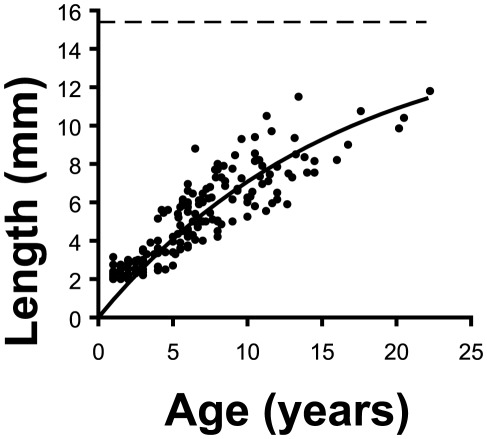
General age-length von Bertalanffy growth curve (see **Eq. 1**) describing the growth in all populations. Dotted line indicates the maximum expected length of corals in all populations (*L_∞_* = 15.4 mm). Points indicate the age/size of all samples in all populations dated by CT scans (*n* = 175) from which the general growth curve was obtained.

*L_∞_* and *K* values (Table 1) were not significantly different among sites (ANCOVA for the slope and intercept of the “Ford-Walford” plot used to estimate *L_∞_* and *K*, *p*>0.05). Age-length data obtained by growth band counts in all the sites were thus merged together, obtaining a general “Ford-Walford” plot to estimate general *L_∞_* and *K* values (Table 1). These parameters produced a general age/length von Bertalanffy growth curve describing growth across all the sampling latitudinal range (Fig. 4).

**Figure 5:**
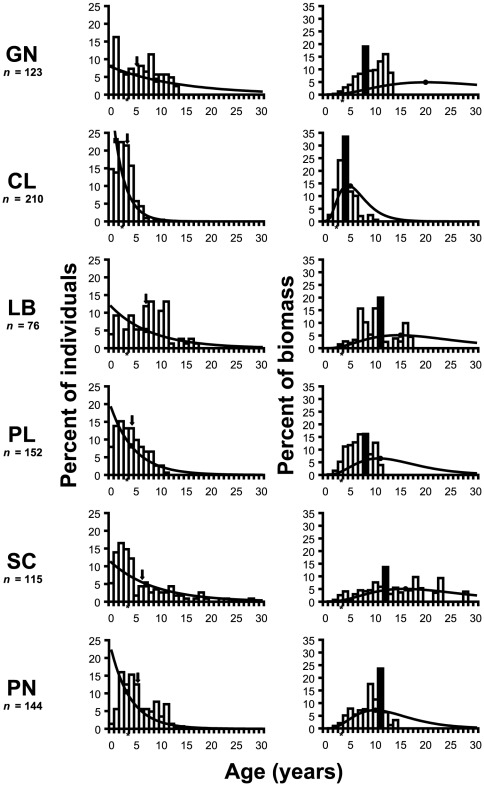
Age class structures of each population. The lines indicate the theoretical distributions. The observed (arrow) and theoretical (black square) age class containing the mean age of the individuals of sampled population are indicated. The observed (black column) and theoretical (black circle) age at maximum percentage biomass are indicated. Asterisks indicate the age at sexual maturity. Data for the Calafuria population (CL) are from [30]. *n* number of individuals dated by growth curves.

All collected individuals in all sites were dated using the general age-length growth curve. The oldest individual came from the Scilla population (SC) with an estimated age of 28 years (12.8 mm length). The age-frequency distributions (Fig. 5) were significantly different among sites (Kolmogorov-Smirnov, *p*<0.001). For each site, a regression of the natural logarithm of the number of individuals (frequency) in each age class (*N_t_*) was computed (Eq. 2). The *r^2^* values of these regressions varied between 0.410–0.838 and were not correlated with SST (Table 1). No observed or theoretical demographic parameter obtained by the analysis of the age-frequency structures was significantly correlated with SST (Table 1 and 2; Fig. 5).

**Table 2:** Correlation analyses between sea surface temperature (independent variable) and demographic parameters (dependent variables) in the sampled populations.

Dependent variable	*n*	*r^2^*	*r*	*r^2^_BS_*	*r_BS_*
Instantaneous rate of mortality (*Z*)	6	0.393	−0.627	0.193	−0.439
Population structure stability	6	0.018	−0.134	0.002	−0.046
Observed % of individuals below sexual maturity	6	0.038	0.195	0.027	0.163
Theoretical % of individuals below sexual maturity	6	0.295	−0.543	0.242	−0.492
Observed mean age	6	0.209	0.457	0.132	0.363
Theoretical mean age	6	0.211	0.459	0.149	0.386
Observed age at maximum % biomass	6	0.492	0.701	0.307	0.554
Theoretical mean age at maximum % biomass	6	0.318	0.564	0.199	0.446
Observed mean age of biomass	6	0.397	0.630	0.264	0.514
Theoretical mean age of biomass	6	0.411	0.641	0.214	0.463

No correlation resulted significant. *n* number of populations. *r^2^*Pearson's coefficient of determination, *r* Pearson's correlation coefficient, *r^2^_BS_* and *r_BS_* Pearson's coefficient calculated with bootstrapping.

## Discussion

The 78–94% of the variance of seawater temperature at the depth of coral sampling (16 m) was explained by variations in SST, indicating that SST generally follows the actual temperature trend present at the sampling depth, as shown by the temperature trends at both depths (Fig. S1). At the Calafuria site, were a full year (November 2001–November 2002) of measurements were available, 84% of SST variance was explained by variations of temperature at 16 m (Fig. S1). This trend was maintained across the 4 analyzed populations, which include the northern (Genova), coldest (Calafuria) and southern-warmest (Pantelleria) sites were corals were collected, and is likely to be maintained also in Elba and Palinuro, which are in between the other sites. Thus, SST has been used as variable to discriminate thermal differences among sites to allow meaningful comparisons between this study and previous investigations on other Mediterranean scleractinian, all of which correlated biological parameters with SST [16], [34], [36], [40].

Polyps of *L. pruvoti* in all of the studied populations were found to reduce their growth rate with increasing age (determinate growth). Within the mechanical constraints of organism design, the environment plays a key role in determining the maximum size an organism can attain [59]. Growth can also be limited by energetic costs, and when an individual decreases its growth rate and has an upper size limit, the excess energy no longer allocated to growth as the individual gets older can be allocated to other processes such as sediment removal, locomotion, maintenance, and reproduction [60]–[62]. Coral species known to have a determinate growth include colonial octocorals [27], [32], [63], [64] and scleractinians such as the branching *Pocillopora spp.* [65], the massive *Goniastrea aspera* [66], and the free-living *Manicina areolata* [67]. Most species for which a determined growth has been observed are solitary forms, such as many free-living fungiids [19], [20], [68], [69], the free-living deep coral *Flabellum alabastrum* [70], and attached polyps such as *B. elegans*, *Paracyathus stearnsii* [71], *B. europaea* [15], [16], and *L. pruvoti* [30]. In these species, the determinate growth could be due to ageing or to a preferential energetic allocation towards processes other than growth, such as reproduction.

Most analyses of coral growth in natural populations are focused on zooxanthellate species, and growth variations are usually related to the varying environmental conditions, such as light intensity, temperature, nutrients and zooplankton [72]. In contrast, knowledge about the growth rates of azooxanthellate corals is very sparse [73]. Measurements of growth in natural populations of azooxanthellate scleractinians are available only for the deep coral *Lophelia pertusa* [74], and for the shallow water *L. pruvoti* at Calafuria [30]. In *L. pruvoti*, the age-length relationships were not significantly different among sites, based on which a general growth curve was obtained (Fig. 4) which describes the growth of individuals across the whole latitudinal range of the present study. Instead, in the Mediterranean endemic solitary zooxanthellate coral *B. europaea*, sampled in the same sites of the present study, during the same time interval, and analyzed using the same methods, the growth constant *K* of the age-length growth curve and the calcification rate are negatively related with SST [16], [40]. The most likely hypothesis to explain the decreasing growth of *B. europaea* with increasing temperature states that the photosynthesis of its symbionts or its calcification machinery would be depressed at temperatures higher than the optimal one for this species, consequently causing an inhibition of calcification [16], [40]. Alternatively, a role may be played by the much steeper response of respiration to subtle temperature increases (Q_10_) than that of photosynthesis, resulting in significant decrease of the residual net photosynthesis and of the energy surplus needed for calcification, growth and reproduction [75]. Besides local factors, the apparent insensitivity of *L. pruvoti* growth to the range of SSTs experienced in the present study may be due either to 1) the lack of zooxanthellate, and thus a lack of inhibition of calcification by the depressed net photosynthesis, or 2) to a higher optimal temperature for the calcification of this species, or 3) a coupling between the above two factors, or 4) a sampling area not representative of the species conditions at the collection sites. However, *L. pruvoti* lives also outside the Mediterranean Sea, up to the southern coasts of Ireland and UK, where seawater temperature is considerably lower [33]. It is then unlikely that this species has a higher optimal temperature for calcification than the Mediterranean endemic *B. europaea*, since *L. pruvoti* lives in much colder seas and deeper waters (up to 70 m depth). The lack of inhibition of calcification by the photosynthetic process seems then the most likely hypothesis to justify the homogeneous growth across the 7 degrees of latitude of the sampling range of the present study. However, any comparison between *L. pruvoti* and *B. europaea* must be taken cautiously, since the two species were sampled at different depths (16 m and 6 m, respectively), which may be subject to different thermal regimes throughout the year.

In theoretical populations with constant mortality across age classes and with a number of recruits equal to the total number of deaths in all age classes (steady state) [76], the coefficient of determination (*r^2^*) of the semi-log regression (Eq. 2) used to estimate the instantaneous rate of mortality (*Z*) is equal to unity [57], [59]. In the sites considered in the present study, *r^2^* varied from 0.8 to 0.4, without a latitudinal trend. This is further emphasized in Table 2, where none of the demographic parameters derived from the age class distributions (which were significantly different among sites) correlated with SST. This indicates that while the growth characteristics are homogeneous across sites, their population dynamics differ, but not according to the SST gradient existing along the sampling latitudinal range. This is quite different to what is reported for the zooxanthellate dendrophylliid *B. europaea* sampled in the same sites of the present study, during the same time interval, and analyzed using the same methods, [16]. *B. europaea* in fact, lives in populations that deviate from the steady state and exhibit a progressive reduction of young individuals with increasing temperature [16]. Even if comparisons between the two species may be biased by the different sampling depths, the variation of population dynamics characteristics among sites, found for *L. pruvoti*, could thus be related to particular local conditions unrelated to temperature. Since the present study focused on the influence of SST, we selected sites with similar environmental traits other than SST, but we did not thoroughly analyzed all the site characteristics such as nutrients and zooplankton availability or competitive interactions with other organisms, which could all contribute to the observed differences in population dynamics traits. However, these local differences, while contributing to the variability of population dynamic characteristics, are not strong enough to determine significant variations in population abundance, which is homogeneous across all sites with about 10,000 individuals per square meter [34]. It may be argued that no correlation with SST has been found because the selected sampling area for this study was too small and unrepresentative of the population. However, the same sampling area adequately represents the sites in previous studies on the biometry and growth of the species, where trends in the biometric parameters (such as polyp length) with temperature have been found [30], [34]. The number of coral specimens collected in the sampling area of the present study (76–210 polyps per site; table 1) is even higher than the one observed in other studies on growth and demography of Mediterranean solitary corals (38–95 polyps per site) where larger sampling areas (1 m^2^) were used because of the lower population density of the species [30], [34], [40]. Moreover, significant differences in the demographic traits among sites have actually been found in the present study, but they do not correlate to temperature, and are likely due to local differences in parameters other than temperature. An alternative explanation of the difference in demographic parameters among sites may be related to suspension feeding. In the Mediterranean, the warm summer–fall season is characterized by lower nutrient levels and zooplankton availability than the cool winter–spring season [77]. Corals and several benthic suspension feeding taxa have proved to be stressed by low nutrients and limited zooplankton availability [77]. Different availability of resources among sites may produce the different demographic traits in *L. pruvoti*. However, if this was the case, negative effects on demographic traits would be expected in the warmest sites (where the warm season is longer and the zooplankton availability lower, on average). Instead, *L. pruvoti* demography seems to be unrelated to SST ([34] and this study). Other environmental parameters not considered in this study may influence coral population dynamics (pH, total alkalinity, wave exposition, flow rate, etc.) and contribute to producing the observed trends. Further investigations are needed to better constrain the environmental controls on the population dynamics of this species, possibly with a higher number of patches per site to better characterize the population dynamics traits at each location.

Global temperature increase is one of the greatest threats for coral and coral reefs survival [78]. The speeds of many negative changes to the oceans are near or are tracking the worst-case scenarios from the IPCC and other predictions [79]. Recently, the coralligenous community, one of the most diverse in the Mediterranean Sea (∼1,666 species) [80] where suspension feeders are dominant, has been strongly affected by several mass mortality events related to high temperatures [81]–[85]. The zooxanthellate dendrophylliid *B. europaea* is a Mediterranean endemic species which is likely to be negatively affected by seawater warming, since increasing temperature lowers its population abundance, its skeletal density [34], by increasing its skeletal porosity [36], and lowers its calcification rate [40]. Moreover, warmer populations are less stable and show a progressive deficiency of young individuals, so that there is concern for the future of this species [16]. All these effects of temperature increase seem to be related to the symbiosis with zooxanthellae, whose photosynthesis could be depressed at high temperatures causing cascading negative effects on the growth and reproductive traits of this species, although this hypothesis is yet to be tested [16], [34], [36], [40]. *L. pruvoti*, instead, seems to be quite tolerant to the same temperature range experienced by *B. europaea*, since none of its biological traits studied until now in the same sites, time interval and using the same methods, results negatively correlated with SST ([34], [36] and present study). It even seems that *L. pruvoti* may benefit from increasing temperature, since corals living in sites with higher SSTs have a higher density of the crystals of calcium carbonate (micro-density) [35] composing their skeleton [36]. However, the limit of temperature increase that will still be tolerable by this species is unknown, and it must be kept in mind that the sampling depth of the two species is different, thus the in situ temperature may be different at the same SST value. These findings indicate that two species belonging to the same family and sharing a wide part of their distribution area may have very different temperature tolerance and consequent response to seawater warming. The higher tolerance of *L. pruvoti*, relative to *B. europaea*, may indeed rely on the absence of symbionts, and thus the lack of an inhibition of host physiological processes by the heat-stressed zooxanthellae. Increasing research effort on determining the effects of temperature on the poorly studied azooxanthellate scleractinians may thus shed light on the possible species assemblage shifts that are likely to occur during the current century as a consequence of global climatic change.

## Supporting Information

Figure S1 **Comparison between SST and temperature at 16 m depth during the indicated time interval.** The left column shows correlation analyses between average daily SST and temperature at 16 m (left column) in the sampling sites for the indicated time interval. The right column shows SST (red line) and temperature at 16 m depth (black line) trends. *n* number of samples (days)
(EPS)

## References

[1] Hoegh-Guldberg O, Dubinsky Z, Stambler N (2011). The impact of climate change on coral reef ecosystems.. Coral reefs: an ecosystem in transition.

[2] Solomon S, Qin D, Manning M, Chen Z, Marquis M (2007). Climate change 2007: the physical science basis.

[3] Richardson AJ, Poloczanska ES (2008). Under-resourced, under threat.. Science.

[4] Walther GR, Post E, Convey P, Menzel A, Parmesan C (2002). Ecological responses to recent climate change.. Nature.

[5] Lejeusne C, Chevaldonné P, Pergent-Martini C, Boudouresque CF, Pérez T (2010). Climate change effects on a miniature ocean: the highly diverse, highly impacted Mediterranean Sea.. Trends Ecol Evol.

[6] Queguiner J (1978). The Mediterranean as a maritime trade route.. Ocean Manage.

[7] Tsimplis MN, Zervakis V, Josey S, Peeneva E, Struglia MV, Lionello P, Malanotte-Rizzoli P, Boscolo R (2006). Changes in the oceanography of the Mediterranean Sea and their link to climate variability.. Mediterranean climate variability.

[8] Grigg RW (1975). Age structure of a longevous coral: a relative index of habitat suitability and stability.. Am Nat.

[9] Loya Y (1976). The red sea coral *Stylophora pistillata* is an r strategist.. Nature.

[10] Buddemeier RW, Kinzie RA (1976). Coral growth.. Oceanogr Mar Biol Annu Rev.

[11] Bablet JP (1985). Report on the growth of a scleractinian.. Proc 5^th^ Int Coral Reef Symp.

[12] Hughes TP, Jackson JBC (1985). Population dynamics and life histories of foliaceous corals.. Ecol Monogr.

[13] Bak RPM, Meesters EH (1998). Coral population structure: the hidden information of colony size-frequency distributions.. Mar Ecol Prog Ser.

[14] Meesters WH, Hilterman M, Kardinaal E, Keetman M, de Vries M (2001). Colony size–frequency distributions of scleractinian coral populations: spatial and interspecific variation.. Mar Ecol Prog Ser.

[15] Goffredo S, Mattioli G, Zaccanti F (2004). Growth and population dynamics model of the Mediterranean solitary coral *Balanophyllia europaea* (Scleractinia, Dendrophylliidae).. Coral Reefs.

[16] Goffredo S, Caroselli E, Mattioli G, Pignotti E, Zaccanti F (2008). Relationships between growth, population structure and sea surface temperature in the temperate solitary coral *Balanophyllia europaea* (Scleractinia, Dendrophylliidae).. Coral Reefs.

[17] Connell JH, Jones OA, Endean R (1973). Population ecology of reef building corals.. Biology and geology of coral reefs, vol. II: biology 1.

[18] Rinkevich B (1995). Restoration strategies for coral reefs damaged by recreational activities—the use of sexual and asexual recruits.. Restor Ecol.

[19] Chadwick-Fuman NE, Goffredo S, Loya Y (2000). Growth and population dynamic model of the reef coral *Fungia granulosa* Kluzinger, 1879 at Eilat, northern Red Sea.. J Exp Mar Biol Ecol.

[20] Goffredo S, Chadwick-Furman NE (2003). Comparative demography of mushroom corals (Scleractinia, Fungiidae) at Eilat, northern Red Sea.. Mar Biol.

[21] Babcock RC (1991). Comparative demography of three species of scleractinian corals using age- and size-dependent classifications.. Ecol Monogr.

[22] Lins de Barros MM, Pires DO (2006). Colony size-frequency distributions among different populations of the scleractinian coral *Siderastrea stellata* in Southwestern Atlantic: implications for life history patterns.. Braz J Oceanogr.

[23] Nozawa Y, Tokeshi M, Nojima S (2008). Structure and dynamics of a high-latitude scleractinian coral community in Amakusa, southwestern Japan.. Mar Ecol Prog Ser.

[24] Harper JL (1977). Population biology of plants.

[25] Hughes RN (1989). A functional biology of clonal animals.

[26] Grigg RW (1984). Resource management of precious corals: a review and application to shallow water reef building corals.. P S Z N I Mar Ecol.

[27] Goffredo S, Lasker HR (2006). Modular growth of a gorgonian coral can generate predictable patterns of colony growth.. J Exp Mar Biol Ecol.

[28] Knuston DW, Buddemeier RW, Smith SV (1972). Coral chronometers: seasonal growth bands in reef corals.. Science.

[29] Logan A, Anderson IH (1991). Skeletal extension growth rate assessment in corals, using CT scan imagery.. Bull Mar Sci.

[30] Goffredo S, Caroselli E, Mattioli G, Zaccanti F (2010). Growth and population dynamic model for the non-zooxanthellate temperate solitary coral *Leptopsammia pruvoti* (Scleractinia, Dendrophylliidae).. Mar Biol.

[31] Ross MA (1984). A quantitative study of the stony coral fishery in Cebu, Philippines.. P S Z N I Mar Ecol.

[32] Goffredo S, Lasker HR (2008). An adaptive management approach to an octocoral fishery based on the Beverton-Holt model.. Coral Reefs.

[33] Zibrowius H (1980). Les scléractiniaires de la Méditeranée et de l'Atlantique nord-oriental.. Mem Inst Oceanogr (Monaco).

[34] Goffredo S, Caroselli E, Pignotti E, Mattioli G, Zaccanti F (2007). Variation in biometry and population density of solitary corals with solar radiation and sea surface temperature in the Mediterranean Sea.. Mar Biol.

[35] Barnes DJ, Devereux MJ (1988). Variations in skeletal architecture associated with density banding in the hard corals *Porites*.. J Exp Mar Biol Ecol.

[36] Caroselli E, Prada F, Pasquini L, Nonnis Marzano F, Zaccanti F (2011). Environmental implications of skeletal micro-density and porosity variation in two scleractinian corals.. Zoology.

[37] Goffredo S, Airi V, Radetić J, Zaccanti F (2006). Sexual reproduction of the solitary sunset cup coral *Leptopsammia pruvoti* (Scleractinia, Dendrophylliidae) in the Mediterranean. 2. Quantitative aspects of the annual reproductive cycle.. Mar Biol.

[38] Goffredo S, Di Ceglie S, Zaccanti F (2009). Genetic differentiation of the temperate-subtropical stony coral *Leptopsammia pruvoti* in the Mediterranean Sea.. Isr J Ecol Evol.

[39] Mundet L, Ribera L (2001). Characteristics of divers at a Spanish resort.. Tour Manag.

[40] Goffredo S, Caroselli E, Mattioli G, Pignotti E, Dubinsky Z (2009). Inferred level of calcification decreases along an increasing temperature gradient in a Mediterranean endemic coral.. Limnol Oceanogr.

[41] Kain JM (1989). The seasons in the subtidal.. Br Phycol J.

[42] Goffredo S, Radetić J, Airi V, Zaccanti F (2005). Sexual reproduction of the solitary sunset cup coral *Leptopsammia pruvoti* (Scleractinia, Dendrophylliidae) in the Mediterranean. 1. Morphological aspects of gametogenesis and ontogenesis.. Mar Biol.

[43] Foster AB, Johnson KG, Schultz LL (1988). Allometric shape change and heterocrony in the freeliving coral *Trachyphyllia bilobata* (Duncan).. Coral Reefs.

[44] Goffredo S, Arnone S, Zaccanti F (2002). Sexual reproduction in the Mediterranean solitary coral *Balanophyllia europaea* (Scleractinia, Dendrophylliidae).. Mar Ecol Prog Ser.

[45] Vermeij MJA (2006). Early life-history dynamics of Caribbean coral species on artificial substratum: the importance of competition, growth and variation in life-history strategy.. Coral Reefs.

[46] Lasker HR (1981). Phenotypic variation in the coral *Montastrea cavernosa* and its effects on colony energetics.. Biol Bull.

[47] Bosscher H (1993). Computerized tomography and skeletal density of coral skeletons.. Coral Reefs.

[48] Helmle KP, Dodge RE, Ketcham RA (2000). Skeletal architecture and density banding in *Diploria strigosa* by X-ray computed tomography.. Proc 9^th^ Int Coral Reef Symp.

[49] Peirano A, Morri C, Bianchi CN (1999). Skeleton growth and density pattern of the temperate, zooxanthellate scleractinian *Cladocora caespitosa* from the Ligurian Sea (NW Mediterranean).. Mar Ecol Prog Ser.

[50] von Bertalanffy L (1938). A quantitative theory of organic growth (inquiries on growth laws II).. Hum Biol.

[51] Fabens AJ (1965). Properties and fitting of the Von Bertalanffy growth curve.. Growth.

[52] Ford E (1933). An account of the herring investigations conducted at Plymouth during the years from 1924–1933.. J Mar Biol Assoc U K.

[53] Walford LA (1946). A new graphic method of describing the growth of animals.. Biol Bull.

[54] Pauly D (1984). Fish population dynamics in tropical waters: a manual for use with programmable calculators.

[55] Sparre P, Ursin E, Venema SC (1989). Introduction to tropical fish stock assessment.

[56] Beverton RJH, Holt SV (1956). A review of methods for estimating mortality rates in fish populations, with special reference to sources of bias in catch sampling.. Rapports et Proces-Verbaux des Reunions - Conseil International pour l'Exploration de la Mer.

[57] Tsounis G, Rossi S, Gili JM, Arntz WE (2007). Red coral fishery at the Costa Brava (NW Mediterranean): case study of an overharvested precious coral.. Ecosystems.

[58] Efron B (1981). Nonparametric estimates of standard error: the jackknife, the bootstrap and other methods.. Biometrika.

[59] Sebens KP (1987). The ecology of indeterminate growth in animals.. Ann Rev Ecol Syst.

[60] Hall VR, Hughes TP (1996). Reproductive strategies of modular organisms: comparative studies of reef- building corals.. Ecology.

[61] Elahi R, Edmunds P (2007). Determinate growth and the scaling of photosynthetic energy intake in the solitary coral *Fungia concinna* (Verril).. J Exp Mar Biol Ecol.

[62] Goffredo S, Caroselli E, Gasparini G, Marconi G, Putignano MT (2011). Colony and polyp biometry and size structure in the orange coral *Astroides calycularis* (Scleractinia: Dendrophylliidae).. Mar Biol Res.

[63] Cordes EE, Nybakken JW, VanDykhuizen G (2001). Reproduction and growth of *Anthomastus ritteri* (Octocorallia: Alcyonacea) from Monterey Bay, California, USA.. Mar Biol.

[64] Bastidas C, Fabricius KE, Willis BL (2004). Demographic aspects of the soft coral *Sinularia flexibilis* leading to local dominance on coral reefs.. Hydrobiologia.

[65] Grigg RW, Maragos JE (1974). Recolonization of hermatypic corals on submerged lava flows in Hawaii.. Ecology.

[66] Sakai K (1998). Effect of colony size, polyp size, and budding mode on egg production in a colonial coral.. Biol Bull.

[67] Johnson KG (1992). Population dynamics of a free-living coral: recruitment, growth and survivorship of *Manicina areolata* (Linnaeus) on the Caribbean coast of Panama.. J Exp Mar Biol Ecol.

[68] Yamashiro H, Nishihira M (1998). Experimental study of growth and asexual reproduction in *Diaseris distorta* (Michelin, 1843), a free-living fungiid coral.. J Exp Mar Biol Ecol.

[69] Knittweis L, Jompa J, Richter C, Wolff M (2009). Population dynamics of the mushroom coral *Heliofungia actiniformis* in the Spermonde Archipelago, South Sulawesi, Indonesia.. Coral Reefs.

[70] Hamel JF, Sun Z, Mercier A (2010). Influence of size and seasonal factors on the growth of the deep-sea coral *Flabellum alabastrum* in mesocosm.. Coral Reefs.

[71] Gerrodette T (1979). Ecological studies of two temperate solitary corals. PhD thesis.

[72] Tambutte S, Holcomb M, Ferrier-Pages C, Reynaud S, Tambutte E (2011). Coral biomineralization: from the gene to the environment.. J Exp Mar Biol Ecol.

[73] Brahmi C, Meibom A, Smith DC, Stolarski J, Auzoux-Bordenave S (2010). Skeletal growth, ultrastructure and composition of the azooxanthellate scleractinian coral *Balanophyllia regia*.. Coral Reefs.

[74] Gass SE, Roberts JM (2006). The occurrence of the cold-water coral *Lophelia pertusa* (Scleractinia) on oil and gas platforms in the North Sea: Colony growth, recruitment and environmental controls on distribution.. Mar Poll Bull.

[75] Al-Horani FA (2005). Effects of changing seawater temperature on photosynthesis and calcification in the scleractinian coral *Galaxea fascicularis*, measured with O_2_, Ca^2+^ and pH microsensors.. Sci Mar.

[76] Wetherall JA, Polovina JJ, Ralston S, Pauly D, Morgan GR (1987). Estimating growth and mortality in steady-state fish stocks from length-frequency data.. Length-based methods in fisheries research. ICLARM conference proceedings.

[77] Coma RM, Ribes M, Gili JM, Zabala M (2000). Seasonality in coastal ecosystems.. Trends Ecol Evol.

[78] Hughes TP, Baird AH, Bellwood DR, Card M, Connolly SR (2003). Climate change, human impacts, and the resilience of coral reefs.. Nature.

[79] Rogers AD, Laffoley Dd'A (2011). International Earth system expert workshop on ocean stresses and impacts. Summary report.

[80] Ballesteros E (2006). Mediterranean coralligenous assemblages: A synthesis of the present knowledge.. Oceanogr Mar Biol Annu Rev.

[81] Cerrano C, Bavestrello G, Bianchi CN, Cattaneo-Vietti R, Bava S (2000). A catastrophic mass-mortality episode of gorgonians and other organisms in the Ligurian Sea (NW Mediterranean), summer 1999.. Ecol Lett.

[82] Perez T, Garrabou J, Sartoretto S, Harmelin JG, Francour P (2000). Mass mortality of marine invertebrates: an unprecedented event in the Northwestern Mediterranean.. C R Acad Sci Paris, Ser III.

[83] Rodolfo-Metalpa R, Bianchi CN, Peirano A, Morri C (2000). Coral mortality in NW Mediterranean.. Coral Reefs.

[84] Coma R, Ribes M, Serrano E, Jimenez E, Salat J (2009). Global warming-enhanced stratification and mass mortality events in the Mediterranean.. Proc Natl Acad Sci U S A.

[85] Garrabou J, Coma R, Bensoussan N, Bally M, Chevaldonne P (2009). Mass mortality in the NW Mediterranean rocky benthic communities: Effects of the 2003 heat wave.. Glob Change Biol.

